# Nutraceutical Supplementation + Holstein Feed Surplus in Rams: Corporal, Metabolic, and Testicular Volumetry-Sperm Variables; The Robin Hood Effect

**DOI:** 10.3390/vetsci13050440

**Published:** 2026-04-30

**Authors:** Ángeles De Santiago-Miramontes, Andrés J. Rodríguez-Sánchez, César A. Meza-Herrera, Ulises Macías-Cruz, Karla Q. Ramírez-Uranga, Cayetano Navarrete-Molina, Pablo Arenas-Báez, Mayela Rodríguez-González, María A. Sariñana-Navarrete, Edgar Díaz-Rojas

**Affiliations:** 1 Graduate Program—Agricultural and Livestock Sciences, Laguna Unit, Antonio Narro Agrarian Autonomous University, Torreon 27054, Coahuila, Mexico; 2 Graduate Program—Natural Resources and Environment in Arid Lands, Regional Universitary Unit on Arid Lands, Chapingo Autonomous University, Bermejillo 35230, Durango, Mexico; 3 Institute of Agricultural Sciences, Autonomous University of Baja California, Mexicali 21705, Baja California, Mexico; 4 Division of Health, Biological and Environmental Sciences, Open and Distance University of Mexico, Ciudad de Mexico 03330, Mexico; 5 Department of Chemical and Environmental Technology, Technological University of Rodeo, Rodeo 35760, Durango, Mexico

**Keywords:** sheep, *Withania somnifera*, Ashwagandha, *Lepidium meyenii*, Maca, Holstein, circular economy

## Abstract

Nowadays, it is central to generate innovations that convert agricultural by-products and food waste into valuable animal products while promoting the long-term resilience and sustainability of vulnerable animal production systems. Nutraceuticals (i.e., ‘nutrition + pharmaceutical’) are derived from foods that offer health benefits. In animal production, nutraceutical supplementation with *Withania somnifera* and *Lepidium meyenii* has shown positive effects on the endocrine, cardiopulmonary, and central nervous systems. We aimed to evaluate the possible impact of nutraceutical supplementation on rams fed a diet based on surplus feed from a highly industrialized Holstein cow production system, on corporal (live weight [LW], kg; body condition score [BCS], units), metabolic (blood glucose [GLU], mg dL^−1^; serum protein [PRO], g 100 mL^−1^), and sexual–testicular variables [sexual odor (ODOR, units); scrotal circumference (SC, cm); testicular volumes (TVOL, cm^3^); and estimated daily sperm production (EDSP, millions)]. Black Belly rams (*n* = 12; LW = 70.36 ± 1.2 kg; BCS = 2.96 ± 0.03 units; age = 3.8 ± 0.2 years; 25° N) were divided into 3 experimental groups: (1) WITH, supplemented with *Withania somnifera* (400 mg kg^−1^ LW d^−1^); (2) LEPI, supplemented with *Lepidium meyenii* (400 mg kg^−1^ LW d^−1^); and (3) CONT, not supplemented. The variables LW, BCS, GLU, PRO, and SC, as well as some components of TVOL, did not differ (*p* > 0.05) among the main effects of treatment or time; only ODOR, right transverse testicular diameter, and total testicular volume differed among treatments, generally favoring the WITH group. Furthermore, the TRT × T interaction demonstrated superior performance (*p* < 0.05) in the WITH group, with the largest values for LW, GLU, PRO, ODOR, SC, width of the right testicle, volume of the right testicle, total testicular volume, and EDSP. From a productive–reproductive perspective, the Robin Hood Effect—through the use of rejected dairy cattle rations as the base diet for rams—and supplemented with nutraceuticals (WITH and LEPI), emerges as a viable alternative to improve not only the productive–reproductive performance of Black Belly rams, but also other productive and socioeconomic outcomes; the latter contributing to the strengthening of producer and family well-being.

## Simple Summary

The use of surplus dairy cow feed rations as the base diet in Black Belly rams, supplemented with the nutraceuticals *Withania somnifera* (WITH) and *Lepidium meyenii* (LEPI), positively influenced the performance of variables of biological and economic importance. Notably, the following response variables showed the highest values in the WITH-group: odor, scrotal circumference, total testicular volume, and estimated daily sperm production. The latter is centered on a Robin Hood effect based on a dairy-cow-intensive system grounded in an extract–produce–dispose strategy rather than a rethink–reuse–reduce approach, mainly observed in less-favored small ruminant production schemes. Such a strategy (i.e., nutraceutical supplementation with surplus Holstein diet) should enhance not only the productive and economic returns of marginal production systems, but also the sustainability, circularity, and well-being of less-favored producers and their families, all within a clean, green, and ethical precision-supplementation framework.

## 1. Introduction

In the last two decades, there has been a growing interest in the use of various nutraceuticals to improve not only production levels but also reproductive performance in zootechnical species under clean, green, and ethical intervention schemes [1,2,3]. While a decline in reproductive outcomes can be exerted by various hormonal, nutritional, and infectious imbalances [4], several nutraceuticals have been evaluated for their ability to improve male reproductive performance [5]. Indeed, the use of nutraceuticals to increase libido and sperm quality has emerged as an alternative therapeutic modality to deal with this scenario [6,7]. Notably, the use of *Lepidium meyenii* (i.e., Maca), a tuber native to the Peruvian Andes, has shown significant effects on sexual health, with improvements in sexual desire, erectile dysfunction, and testicular function, with parallel increases in Leydig cells and testosterone synthesis [8,9,10]. *Lepidium meyenii* is recognized as a functional food and phytogenic supplement with adaptogenic properties whose biological effects are attributed to a complex matrix of bioactive secondary metabolites such as macamides, macaenes, glucosinolates, and alkaloids associated with antioxidant protection, androgenic activity, and the modulation of energy metabolism [11]. They also act as protective agents against heat stress by improving the hormonal profile and mitigating the decline in performance of livestock exposed to extreme temperatures [12]. Furthermore, positive effects have been reported from a therapeutic standpoint following ingestion of the roots of a small evergreen shrub, *Withania somnifera* (i.e., Ashwagandha) [6,13], classified as a phytogenic nutraceutical and adaptogen that acts as a metabolic regulator, increasing the capacity for adaptation to environmental stress. Its therapeutic potential stems from a diverse phytochemical composition characterized by withanolides, sitoindosides, and alkaloids with anti-inflammatory and antioxidant effects [14,15]. These compounds modulate the hypothalamic–pituitary–adrenal (HPA) axis by attenuating cortisol secretion, thereby conferring biological resilience under conditions of thermal and metabolic challenge [14,15]. In Mexico, while the goat population showed a decrease of 7.5% between 1970–2020, the sheep inventory increased by 37%, with a figure of 8.5 million sheep [16]. In the Comarca Lagunera, northern arid Mexico, sheep meat production was close to 180,000 kg, with a production value of 660,000 USD. The productive and economic indicators of sheep for meat production in the Comarca Lagunera are potentially important; during the period 2000–2020, sheep inventory grew by 318%, meat production by 600%, and production value by 580%, which marked the highest growth in sheep inventory and an increased economic spillover generated by hair sheep [16].

From an economic standpoint, reproductive efficiency is essential for increasing the pregnancy rate and consequently reducing the generation interval. Therefore, the reproductive efficiency of the male is central to achieving greater viability not only within the herd, but also from a genetic and economic standpoint [1,2,7,17]. While the testis plays an endocrine role in testosterone production, it also has an exocrine role in producing sperm [18]. In addition, while testicular function is directly correlated with testicular volume—since approximately 98% of testicular volume is composed of seminiferous tubules and germ cells—it is also modulated by nutritional intake [19]. In this regard, and considering the productive context of the Comarca Lagunera, highly industrialized dairy production schemes are characterized by computer-formulated total mixed rations (TMR), mechanized delivery, high stocking densities, and closed-cycle management, which inevitably generate daily feed refusals that retain high nutritional value yet remain largely unused within the conventional linear production model. Moreover, the circular economy approach has emerged as a strategic framework to reduce waste and optimize resource use, in contrast to traditional linear systems of extraction, production, and disposal. In ruminant livestock production, this approach is particularly relevant, as these animals can convert fibrous residues into high-value products. In semiarid regions, where forage, water, and environmental stability are limited, circular economy strategies are essential to strengthen the resilience and sustainability of the system. Thus, building on previous findings, we hypothesize a positive effect of nutraceutical supplementation on certain productive–reproductive variables in rams fed surplus feed from the diet offered to Holstein cows managed under highly industrialized production schemes in the Comarca Lagunera. We aimed to evaluate the possible effect of supplementation with the nutraceuticals *Withania somnifera* and *Lepidium meyenii* with respect to corporal variables (live weight [LW], kg; body condition score [BCS], units), sexual variables (sexual odor [ODOR], units; scrotal circumference [SC], cm; testicular volume [TVOL], cm^3^), and metabolic variables (blood glucose [GLU], mg dL^−1^; serum protein [PRO], g 100 mL^−1^) in Black Belly rams fed a base diet constituted by the surplus of a highly industrialized production system of Holstein cows in northern Mexico (25° N), during May, June, and July, which coincides with a period of significant thermal stress in the region.

## 2. Materials and Methods

All methods, experimental procedures, and management of the experimental units used in this study complied with the guidelines for the ethical use, care, and welfare of animals in research at the international [20] and national [21] levels, while aligned to the Mexican Official Standard NOM-062-ZOO-1999 [22], receiving institutional approval, referenced with the code UAAAN-UL-425502002-2832.

### 2.1. Location, Environmental Conditions and Reproductive Seasons

The study was carried out in a sheep herd managed under semi-intensive conditions (Ejido Granada, Matamoros, Coahuila), in the Comarca Lagunera in northern Mexico, at a latitude of 25° N and longitude 103° W., and an altitude of 1110 m above sea level. The region has an average annual temperature of 23.8 °C, a maximum of 44 °C in May, and a minimum of −1 °C in December and January, with an annual rainfall of 230 mm [23]. Interestingly, May 2025 was the driest and hottest month of the year, with extreme temperatures reaching 44 °C, an average relative humidity of 30%, and minimums and maximums of 17.5% and 46%, respectively. In June and July, there were atypical rains, with relative humidity averages of 40%, 16%, and 58%, respectively [23,24]. The study was carried out during May, June, and July, which involved the transition to breeding (May–June) and the reproductive season (June–July).

### 2.2. Treatments and Variables to be Evaluated

Black Belly rams (*n* = 12), with proven fertility and libido, homogeneous in terms of live weight (LW; 70.36 ± 1.2 kg), body condition score (BCS; 2.96 ± 0.03 units), and age (3.8 ± 0.2 years), were divided in early May into 3 experimental groups (May–July; 63 d): supplemented with *Withania somnifera* (WITH; 400 mg kg^−1^ LW d^−1^), supplemented with *Lepidium meyenii* (LEPI; 400 mg kg^−1^ LW d^−1^), or non-supplemented (CONT). The preparation and administration of both supplements followed the methodology described by Azeez et al. [25]. For *Withania somnifera*, standardized caplets (Himalaya Herbal Healthcare, Bangalore, India), each containing 500 mg of root powder standardized to 5% withanolides, ensuring product quality and concentration consistency throughout the experimental period. For *Lepidium meyenii*, standardized root extract vegetable capsules were used (200 mg capsule^−1^; 5% macamides; Nootropics Depot, Scottsdale, AZ, USA), with a GMP-certified manufacturer with independent third-party laboratory verification. The use of these standardized presentations ensured uniformity in the concentration of plant material used throughout the experimental period. The *W. somnifera* tablets were mechanically ground into a fine homogeneous powder, while the *L. meyenii* capsules were manually opened to release the extracted powder contained within. In both cases, the resulting powder was weighed daily on an individual basis for each ram and adjusted weekly according to changes in body weight, to maintain the established dose of 400 mg kg^−1^ LW. The calculated dose, each animal was dosed individually in the morning (before feeding) via oral drenching; the treatment was administered diluted in 400 mL of distilled water.

The basal diet consisted of the surplus derived from a Total Mixed Ration (TMR) formulated for lactating Holstein cows under intensive production systems in the Comarca Lagunera. The composition of the surplus was determined through periodic sampling (i.e., every 10 days), collected along the feed line, pooled, and subsequently subjected to chemical and physical analysis. The ingredient composition (g kg^−1^ DM) included corn silage (241.6), alfalfa hay (135.2), rolled corn grain (310.7), soybean meal (123.5), cottonseed meal (94.5), and a vitamin-mineral mix (94.5). The analyzed nutrient composition (%) was dry matter (80.0), ash (7.3), ether extract (3.8), crude protein (15.3), neutral detergent fiber (26.9), acid detergent fiber (17.8), starch (30.9), and total digestible nutrients (88.9), with an estimated metabolizable energy content of 3.5 Mcal kg^−1^ DM, and a net energy for lactation (NEL) of 2.06 Mcal kg^−1^ DM. Nutrient composition was determined according to the official methods of AOAC International [26]. Before the initiation of the experimental period, all rams underwent a 21-day adaptation period to the basal diet consisting of the surplus derived from the TMR formulated for lactating Holstein cows. The 12 rams were housed in individual pens; the basal diet was offered twice daily (0700 h and 1600 h) at 3.5% of live body weight. The rejection of the experimental diet, if any, was removed daily before each new allotment. Clean, fresh water was provided ad libitum. Regular cleaning and fly population control were carried out in both the general corrals and the experimental pens. The response variables considered in the study included: Corporal measurements (live weight LW, kg; body condition score BCS, units), Reproductive outcomes (sexual odor ODOR, units; scrotal circumference SC, cm; testicular volumes TVOL, cm^3^), and Metabolic values (blood glucose GLU, mg dL^−1^; serum protein PRO, g 100 mL^−1^); the variables were recorded at different stages of a 63 days experimental period.

1. Corporal variables: Body condition score and live weight. Body condition score (BCS) was recorded by palpation of the thickness of muscle and adipose mass in the space between the spinous and transverse processes of the lumbar vertebrae, with a range of 1 to 4 (1 = emaciated and 4 = obese) according to the technique described by Walkden-Brown et al. [27]. Additionally, the live weight (LW) was recorded using a digital scale with a capacity of 150 kg and an accuracy of at least 50 g (WH-C 100, WeiHeng, Heyuan City, China).
2. Reproductive variables: Sexual odor intensity, scrotal circumference, and testicular volumes. Sexual odor (ODOR) was evaluated based on the technique described by Walkden-Brown et al. [27]; briefly, the back of the base of the horns was smelled at a distance of 15 cm considering four ranges (0 to 3), where (0) is defined as neutral odor or odor equal to females, (1) light ram smell, (2) moderate ram smell, and (3) heavy ram smell. All ODOR evaluations were performed by the same trained observer under consistent environmental and handling conditions to ensure scoring consistency throughout the experimental period. Scrotal circumference (SC) was measured by using a flexible tape measure graduated in millimeters to record the widest point of the testes, following the technique described by Cruz-Castrejon et al. [28]. To quantify the total testicular volume (TTVOL, cm^3^), testicular examinations were performed as follows: the scrotum was supported on a rolled towel placed under the scrotum to stabilize the testicles, and coupling gel was generously applied to minimize interference from the surrounding air. The testes were further stabilized by placing a finger on the median raphe of the scrotal skin before gently scanning to avoid distortion of their shape and dimensions. By using ultrasound equipment (Aloka SSD 500, Aloka Co., Tokyo, Japan), with a high-resolution linear probe of 7.5 MHz, images of the testes were acquired in the longitudinal and transverse planes. Testicular length (WIDTH) and height (HEIGHT) were measured in the longitudinal view, while depth was measured in the transverse view (TRANS), first the right and then the left using the testicular mediastinum as a reference; the epididymis was not included in the measurement. The volume (cm^3^) of each testis (LFT and RIG) was calculated using the formula 0.61 [(width)(height)(trans)] [29]; the sum of both generated the average total testicular volume (TTVOL) per experimental group. To ensure high methodological consistency and minimize experimental error, all ultrasound scans and image interpretations were performed by the same experienced operator using a standardized protocol of longitudinal and transverse planes, guaranteeing technical consistency and intra-observer reliability across treatments. Also, the linear regression proposed by Montes-Garrido et al. [29] was used to calculate the estimated daily sperm production (millions) (i.e., EDSP, Millions): EDSP (M) = [(0.02226 × TTVOL) − 2.977] × 1000.
3. Metabolic variables (glucose and serum protein). To determine serum glucose and protein levels, a blood sample was obtained from the males of each experimental group, collecting 5 mL, by direct puncture of the jugular vein using a red plug tube, which allows blood coagulation (serum). Each sample was centrifuged (15 min 3500× *g*) to separate the serum and was stored at −20 °C until further analysis. While serum total protein (PRO) concentrations were determined in duplicate using a commercial bicinchoninic acid reagent-based kit considering bovine serum albumin 16 as standard and performed as described in the manual kit (Pierce Chemical Co., Dallas, TX, USA), serum glucose (GLU) analyses were performed in duplicate via spectrophotometer techniques, following the protocols provided by the kit manufacturer (Roche Diagnostic Systems, Inc., Indianapolis, IN, USA). Samples were obtained from each male within and among experimental groups under fasting conditions.

### 2.3. Statistical Analyses

The corporal, metabolic, testicular-morphometric, and sperm-production response variables throughout the experimental period (i.e., across time) were analyzed by linear mixed models for repeated measures in the same ram across time through the PROC MIXED procedure. While the nutraceutical supplementation was defined as the fixed effect, the repeated measures were the sampling period. Each sampling period was expected to represent the diverse measures of diverse response variables of the same ram under different time conditions; thus, the ram within the supplementation class was assumed to be the random effect. Because the experimental period across time considered different measurement spots, the spatial power covariance structure [*SP(POW)*] was used in the analysis; the containment approximation was considered to calculate the denominator degrees of freedom. When the data were equally spaced, spatial power yielded non-different results as a first-order autoregressive structure [*AR(1)*]. All data were normally distributed and presented as LS means ± standard error of the mean (SEM) for the studied parameters; the only non-normally distributed variable was GLU, which was log-transformed to reduce skewness, yet the non-transformed values are reported. Since both supplementation and evaluation–quantification of variables were carried out individually in each ram, each male was considered the experimental unit. In the event of a significant effect, the media separation considered the LSMEAN/PDIFF option of the PROC GLM. All statistical analyses were performed using the SAS statistical package (SAS Inst. Inc., Ver. 9.4, 2016, Cary, NC, USA). The results are expressed as least square means; statistical differences were considered at *p* < 0.05.

## 3. Results

### 3.1. Effect of Supplementation on the Response Variables Analyzed

While Table 1 shows the results obtained for the dependent variables: LW (kg), BCS (units), GLU (mg dL^−1^), and PRO (g 100 mL^−1^), Table 2 concentrates the information for the same response variables but across time; no differences (*p* > 0.05) occurred either among treatments or across time.

Table 3 shows the means by TRT of reproductive variables SC (cm); ODOR (units), and testicular volumetry i.e., length (WIDTH, cm); height (HEIGHT, cm); and depth (TRANS, cm) either left (LFT) or right (RIG). Two volumes (i.e., TVOL) for each testicle (i.e., LFT and RIG), the average of the total testicular volume (TTVOL, cm^3^), as well as the estimated daily sperm production (EDSP, millions) per male, were obtained. Of the indicated variables, ODOR, TRANS-RIG, and TTVOL differed (*p* < 0.05) among treatments during the experimental period. The highest ODOR value occurred in WITH, followed by LEPI and CONT, observing a variation of 77.8% between the extreme values; TRANS-RIG showed a similar trend. Regarding TTVOL, the highest value was observed in WITH, and the lowest in LEPI, with a 17.3% difference between them, favoring the WITH group.

### 3.2. Effect of the Supplementation x Time Interaction on the Response Variables Analyzed

The interaction analysis between the type of supplementation and the experimental period showed differences (*p* < 0.05) in the variables LW, BCS, GLU, and PRO (Table 4). While the LW showed an upward trend in WITH and LEPI, the increase in LW at the end of the experimental period was negligible in CONT. In general, the BCS remained constant between LEPI and CONT throughout the study, except for WITH in the initial period, observing the lowest value regarding time and treatments, although without differences in the middle and final stages of the study. The metabolic variables showed unstable performance throughout the experimental period. The performance of the treatment × time interaction for reproductive variables (ODOR, units; SC, cm), testicular volumetry [length (WIDTH, cm), height (HEIGHT, cm) and depth (TRANS, cm) in the left (LFT) and the right (RIG) testicle], the individual testicular volume (i.e., TVOL; LFT and RIG), the average total testicular volume (TTVOL, cm^3^), and the estimated daily sperm production (EDSP, millions) are concentrated in Table 5. The effect of the TRT × T interaction affected the performance of: ODOR, SC, WIDTH-LFT, WIDTH-RIG, TVOL-RIG, TTVOL, and EDSP. In general, the volumetric testicular variables favored the WITH group, observing the highest values towards the end of the experimental period. Although all the variables analyzed are important, it is central to highlight that the variables odor, scrotal circumference, total testicular volume, and estimated daily sperm production showed the highest values in the group supplemented with *Withania somnifera*.

## 4. Discussion

The results obtained support our working hypothesis, as there is a treatment × time interaction effect in the analyzed response variables in Black Belly rams fed a base diet composed of feed waste from a highly industrialized production system of Holstein cows, and supplemented with nutraceuticals (i.e., WITH and LEPI); this response was evident even under the high environmental heat load typical of the study area during May, June, and July. Although ODOR was the only variable that differed due to main effects (i.e., treatment and time), most of the dependent variables differed with respect to the treatment × time interaction (i.e., TRT × T). Moreover, the response variables odor, scrotal circumference, total testicular volume, and estimated daily sperm production showed the highest values in the group supplemented with *Withania somnifera*. This is particularly relevant given that the response variables are central from both productive and reproductive perspectives [30]. In sheep, other studies have reported differences in corporal, productive, and reproductive variables [30,31,32,33,34]. Our results indicate beneficial effects of WITH supplementation, whose active ingredients promote growth and modulate anti-stress, antioxidant, immunomodulatory, and hepatoprotective actions [35,36,37,38]. In general, the LEPI group showed better performance compared to the CONT group, possibly due to its effects on energy metabolism, thermoregulation, and electrolyte balance [39,40], as well as physical resistance and thermal mitigation [41], coupled with its thermoregulatory effect in mammals [10,42]. The present study demonstrates that supplementation with WITH and LEPI over time improved various variables of productive and reproductive importance in Black Belly rams fed a base diet of surplus feed ration for dairy cows in the study area. The mechanisms and sites of action of supplemented nutraceuticals (i.e., WITH and LEPI) on corporal, metabolic, and reproductive variables cannot be fully elucidated without complementary research, particularly at the physiological and molecular levels. Although a larger sample size would strengthen the inferences, the strict phenotypic homogeneity of the experimental units and the repeated-measures design allowed for the identification of clear biological trends. These findings establish an initial, preliminary physiological characterization to justify future trials involving advanced sperm analyses (i.e., CASA).

The response variables ODOR, TRANS-RIG, TTVOL, and EDSP were the only ones that differed among treatments, with the highest values observed in the WITH group, followed by LEPI for ODOR and TRANS-RIG, and by CONT for TTVOL and EDSP. These results are particularly relevant since corporal variables are essential traits in animal production, serving as criteria for phenotypic selection and economic profitability [43]. The positive effects of WITH supplementation on LW phenotypic expression have been reported in various species [44,45,46] and are noteworthy given that body traits such as LW and BCS affect growth characteristics [31,47]. Regarding testicular morphometry, SC is positively correlated with reproductive capacity; producers consider testicular features when selecting replacement animals [48]. The size difference between the left and right testes observed in the present study, even in the control group, is consistent with the well-documented biological characteristics of rams. Karimi et al. [49] pointed out that in ruminants, the right and left testes often differ in size and position and are frequently unequal in size. Interestingly, the high degree of symmetry observed in the WITH and LEPI groups (<2% difference) suggests that these nutraceuticals may help maintain morphological stability even under environmental stress. *Withania somnifera* (i.e., WITH) has anti-inflammatory, antitumor, anti-stress, and antioxidant effects; it acts as an immunomodulator and improves hematopoiesis, in addition to exerting possible effects on the endocrine, cardiopulmonary, and central nervous systems [6]. The sperm quality of infertile males can be improved by oral intake of *Withania somnifera* root; it inhibits lipid peroxidation and improves sperm count and motility, while also regulating reproductive hormone levels, with additional benefits for overall health status [2,13]. Furthermore, the positive effect of LEPI supplementation has been attributed to its action on energy metabolism, thermoregulation, and electrolyte balance [39,40], as well as physical resistance and mitigation of heat stress [40]; its mineral content has been involved in electrolyte balance, particularly during periods of increased physical activity and heat exposure [39,50,51]. Although our study did not include physical challenges, the animals were subjected to excessive solar radiation and heat exposure arising from the natural climatic conditions of arid environments. This context is important, as temperatures reaching 44 °C constitute a severe thermal insult capable of inducing oxidative stress and testicular hypoplasia. Our findings suggest that the antioxidant and adaptogenic properties of WITH and LEPI acted as a physiological buffer against these extreme conditions. In this regard, evidence from male ruminants indicates that sustained heat stress-driven cortisol elevation suppresses the HPG axis through inhibition of GnRH pulsatility and reduced gonadotropic sensitivity at the pituitary level, directly impairing Leydig cell steroidogenesis and spermatogenic activity [52,53,54]. The main bioactive compounds of *Withania somnifera* are withanolides—a family of steroidal lactones—among which the most relevant for reproductive and metabolic function are: Withaferin A (antioxidant and anti-inflammatory activity), Withanolide A (neuroprotective and modulator of reproductive function), and Withanone (cytoprotective activity). *Withania somnifera* appears to have counteracted this mechanism through two complementary pathways: centrally, by attenuating HPA hyperactivation and thereby preserving the gonadotropic support necessary for testosterone synthesis in Leydig cells [55,56]; and peripherally, by upregulating the Nrf2/HO-1 cytoprotective pathway and downregulating NF-κB expression in testicular and epididymal tissues, thereby reducing lipid peroxidation and mitigating germ cell apoptosis under chronic oxidative stress [57,58]. In turn, the main bioactive compounds of *Lepidium meyenii* are macamides—N-benzylamide derivatives of long-chain fatty acids—among which the most relevant for reproductive function are: N-benzylhexadecanamide (the most abundant macamide, associated with improvements in sperm count and motility), N-benzyl-(9Z,12Z)-octadecadienamide (positive impact on seminal parameters), and N-benzyl-(9Z,12Z,15Z)-octadecatrienamide (antioxidant and anti-inflammatory peripheral activity). *Lepidium meyenii*, in contrast, does not appear to modify serum concentrations of LH, FSH, or testosterone under basal conditions [59], suggesting that its reproductive benefits under heat stress are mediated primarily through peripheral antioxidant pathways—scavenging reactive oxygen species via macamides, glucosinolates, and alkaloids, improving total antioxidant capacity, and reducing cortisol-mediated oxidative damage [12,60]—rather than through central neuroendocrine regulation, as observed with WITH. This mechanistic divergence is consistent with the differential glycemic responses documented between groups and suggests that both nutraceuticals operate through fundamentally distinct regulatory nodes. While the CONT group showed a trend toward reduced testicular development, likely due to heat-induced parenchymal impairment, the supplemented rams maintained superior testicular volume and architecture. This indicates that the nutraceuticals did not merely promote growth but actively mitigated the negative effects of the high environmental heat load, preserving the physiological integrity required for sperm production. Specifically, the decrease in total testicular volume observed in the CONT and LEPI groups toward the end of the study reflects the impact of extreme environmental temperatures (>41 °C) in the Comarca Lagunera, which can override seasonal photoperiodic stimuli and induce temporary testicular regression [61,62]. Conversely, the volume increase in the WITH group aligns with the adaptogenic and gonadotropic properties of *W. somnifera*, which has been shown to enhance testicular development and seminiferous tubule diameter even under suppressive conditions [63]. The TRT × T interaction affected phenotypic performance across all response variables analyzed. The time-dependent metabolic responses observed in GLU and PRO are consistent with the adaptogenic and cumulative nature of both nutraceuticals, whose mechanisms of action require sustained exposure to become physiologically detectable. Regarding glucose, the WITH group maintained the highest values throughout the study, consistent with findings reported by Bhattacharya et al. [64], Pedhavi et al. [65], and Razo et al. [66], while the LEPI group showed the lowest blood glucose levels, in line with the reported regulatory effects of LEPI on energy metabolism and its associated antioxidant activity [67,68,69,70]. Regarding serum protein, the WITH group showed a favorable trend in PRO concentrations over time, consistent with the effects reported by Pedhavi et al. [65] and Razo et al. [66] in sheep supplemented with WITH, an effect associated with the phenolic and tannin content characteristic of this adaptogen [71,72,73]. The relatively lower PRO values observed in the LEPI group toward the end of the study are consistent with previously documented patterns in biological models supplemented with LEPI extracts [74,75], and likely reflect the differential metabolic regulatory pathways through which each nutraceutical operates under conditions of sustained thermal challenge.

Beyond the biological results, another noteworthy aspect is the circular economy strategy on which our research was based, considering the use of a base diet for sheep feed derived from the surplus of the Holstein dairy industry in the Comarca Lagunera, regarded as the main dairy cattle hub in Mexico. Sheep meat production in the study area, although negligible compared to the dairy cattle industry, holds a socioeconomic relevance that should not be underestimated. Indeed, considering a circular economy approach, the surpluses of the dairy industry have the potential to boost sheep meat production in the region through a strategy of minimizing production costs. When evaluating 50 intensive dairy cattle farms, a daily average of 21.42 kg (wet basis) was offered per milked cow (MC^−1^ d^−1^), along with an average dry matter of 84.45% (18.09 kg DM^−1^, dry basis) and a rejection rate of 4.5%. This surplus is equivalent to 0.96 kg MC^−1^ d^−1^ on a wet basis. Therefore, a census of 264,638 milked cows [16] would generate a daily availability of 254.05 tons (wet basis) and 215.4 tons (dry basis), considering feed refusals from dairy cattle under this industrialized production scheme.

Avendaño and Navarro [76] reported that ewes across various physiological stages (mean body weight [BW]: 41.82 kg) exhibit a daily dry matter intake (DMI) equivalent to 2.88% of their body weight. Based on these estimates, the nutritional potential of feed waste recovered from the dairy cattle industry could sustain approximately 180,000 head of sheep [76]. Implementing this “Robin Hood Effect” [30,77,78] would allow for an 18-fold increase in the sheep population within the Comarca Lagunera region, Mexico. Considering an average carcass yield of 24.28 kg per animal [16], this system could generate 4343.56 tonnes of mutton. Consequently, the upcycling of dairy industry by-products can enhance livestock production in arid regions while mitigating nutritional and economic pressures on vulnerable farming systems. The socioeconomic impact of the “Robin Hood Effect”—the strategic utilization of dairy feed waste for sheep production—is substantial. At an international market price of 5778.37 USD per tonne of meat [79], this circular economy strategy could generate an annual economic input of 25.10 million USD in the Comarca Lagunera. Furthermore, based on established economic contribution margins in Mexico, ranging from 13.3% to 30.6% [80,81,82], this model would directly benefit between 8914 and 20,059 households. Given the acute water crisis in northern Mexico and prevailing social inequalities [83], this circular strategy represents an innovative and sustainable approach that aligns the livestock sector with the 2030 Agenda for Sustainable Development [84].

## 5. Conclusions

Supplementation with *Withania somnifera* (WITH) in Black Belly rams fed a dairy feed surplus basal diet improved productive, metabolic, and reproductive responses (specifically LW, GLU, PRO, ODOR, SC, RIG, TTVOL, and EDSP), outperforming *Lepidium meyenii*. This model demonstrates high socioeconomic potential, generating 4343.56 tonnes of meat and an annual economic impact of 25.10 million USD, directly benefiting up to 20,059 rural households. The “Robin Hood Effect” shifts the intensive “extract–produce–dispose” strategy toward a “rethink–reuse–reduce” approach, enhancing productive returns and well-being in marginal production systems. Future research should replicate this study with larger sample sizes and further explore the physiological and molecular mechanisms of action, to consolidate these findings within a framework of sustainability and circular economy.

## Figures and Tables

**Table 1:** Least square means by treatment of corporal variables live weight, (LW, kg), body condition score, (BCS, units) and metabolic variables blood glucose (GLU), and serum protein (PRO) in Black Belly sheep (n = 12) supplemented with *Withania somnifera* (WITH; 400 mg kg^−1^ LW d^−1^), *Lepidium meyenii* (LEPI; 400 mg kg^−1^ LW d^−1^), or non-supplemented (CONT) during May–July (64 d) in northern Mexico (25° N) ^1^.

Variable	WITH	LEPI	CONT	SEM ^2^	*p*-Value
LW (kg)	71.5	71.4	68.2	1.2	0.19
BCS (units)	2.9	3.0	3.0	0.0	0.42
GLU (mg dL^−1^)	51.0	45.0	48.8	2.2	0.23
PRO (g 100 mL^−1^)	7.5	7.4	7.4	0.3	0.93

^1^ No differences (*p* > 0.05) among least square mean within variables across treatments, occurred. ^2^ The most conservative standard error of the mean (SEM) is presented.

**Table 2:** Least square means across time for corporal variables [live weight (LW); body condition score (BCS)] and metabolic variables [blood glucose (GLU), serum protein (PRO)] in Black Belly sheep (n = 12) supplemented with *Withania somnifera* (WITH; 400 mg kg^−1^ LW d^−1^), *Lepidium meyenii* (LEPI; 400 mg kg^−1^ LW d^−1^), or non-supplemented (CONT) during May–July (64 d) in northern Mexico (25° N) ^1^.

Variable/Day	0	10	20	30	40	50	63	SEM ^2^	*p*-Value
LW (kg)	68.1 ^2^	69.1	71.1	70.9	70.7	71.2	71.5	1.9	0.62
BCS (units)	3.0	3.0	3.0	3.0	3.0	3.0	3.0	0.0	0.93
GLU (mg dL^−1^)	49.9	48.4	48.3	41.7	48.4	50.9	49.9	3.4	0.35
PRO (g 100 mL^−1^)	7.2	7.3	7.2	7.0	7.7	7.8	7.2	0.4	0.82

^1^ No differences (*p* > 0.05) among least square means within variables across time, occurred. ^2^ The most conservative standard error of the mean (SEM) is presented.

**Table 3:** Least square means by treatment of reproductive and morphometric-testicular variables in Black Belly sheep (n = 12) supplemented with *Withania somnifera* (WITH; 400 mg kg^−1^ LW d^−1^), *Lepidium meyenii* (LEPI; 400 mg kg^−1^ LW d^−1^), or non-supplemented (CONT) during May–July (64 d) in northern Mexico (25° N).

Variables ^1^	WITH	LEPI	CONT	SEM ^2^	*p*-Value
ODOR (units)	0.9 ^a^	0.7 ^b^	0.2 ^c^	0.1	0.01
SC (cm)	34.8 ^a^	33.8 ^a^	33.7 ^a^	0.6	0.84
WIDTH-LFT (cm)	5.1 ^a^	4.8 ^a^	5.4 ^a^	0.2	0.82
WIDTH-RIG (cm)	5.2 ^a^	4.3 ^a^	4.3 ^a^	0.2	0.80
HEIGHT-LFT (cm)	8.9 ^a^	9.3 ^a^	9.2 ^a^	0.2	0.59
HEIGHT-RIG (cm)	9.3 ^a^	8.5 ^a^	9.0 ^a^	0.7	0.63
TRANS-LFT (cm)	4.4 ^a^	4.4 ^a^	4.5 ^a^	0.1	0.89
TRANS-RIG (cm)	4.9 ^a^	4.3 ^a,b^	4.0 ^b^	0.4	0.72
TVOL-LFT (cm^3^) ^3^	121.8 ^a^	119.8 ^a^	136.4 ^a^	5.9	0.61
TVOL-RIG (cm^3^)	144.5 ^a^	95.9 ^a^	94.4 ^a^	14.2	0.59
TTVOL (cm^3^)	266.4 ^a^	215.7 ^b^	230.8 ^a,b^	14.2	0.04
EDSP (million) ^4^	2996.8 ^a^	1802.8 ^b^	2288.4 ^b^	262.4	0.02

^1^ Sexual odor (ODOR, units); scrotal circumference (SC, cm), testicular volumetry (length (WIDTH, cm); height (HEIGHT, cm); depth (TRANS, cm) for left (LFT) and right (RIG) testicles, individual (i.e., TVOL cm^3^, left and right), and total testicular volumes (TTVOL cm^3^), as well as the estimated daily sperm production (EDSP, millions). ^2^ The most conservative standard error of the mean (SEM) is presented. ^3^ The calculation of the individual testicular volume (cm^3^) considered the formula 0.61 [width × height × trans] (i.e., TVOL; LFT and RIG). The sum of both generated the average total testicular volume (TTVOL); The epididymis was not included in the measurement. ^4^ The estimated daily sperm production (millions) considered the linear regression EDSP (M) = [(0.02226 × TTVOL) − 2.977] × 1000. ^a–c^ Least square means with different superscripts within variables among treatments differ (*p* < 0.05; PROC GLM, PDIFF option).

**Table 4:** Least square means of the treatment × time interaction of corporal variables live weight, (LW, kg), body condition score, (BCS, units) and metabolic variables blood glucose (GLU), and serum protein (PRO) in Black Belly sheep (n = 12) supplemented with *Withania somnifera* (WITH; 400 mg kg^−1^ LW d^−1^), *Lepidium meyenii* (LEPI; 400 mg kg^−1^ LW d^−1^), or non-supplemented (CONT) at different stages of the experimental period (May–July, 64 d) in northern Mexico (25° N).

Variable/Period	WITH			LEPI			CONT			
	Initial	Middle	Final	Initial	Middle	Final	Initial	Middle	Final	*p*-Value
LW (kg)	68.4 ^c–f^	72.2 ^a–d^	73.0 ^a,b^	68.7 ^b–f^	72.0 ^a–d^	72.9 ^a,b^	67.0 ^f^	68.4 ^c–f^	68.6 ^c–f^	0.01
BCS (units)	2.8 ^b^	3.0 ^a^	3.0 ^a^	3.0 ^a^	3.0 ^a^	3.0 ^a^	3.0 ^a^	3.0 ^a^	3.0 ^a^	0.04
GLU (mg dL^−1^)	51.0 ^a^	46.3 ^a,b,c^	55.6 ^a^	44.6 ^a,b,c^	38.0 ^d^	48.3 ^a,b,c^	54.0 ^a^	40.6 ^c,d^	45.6 ^a,b,c^	0.01
PRO (g 100 mL^−1^)	7.1 ^b^	7.6 ^a^	7.6 ^a^	7.2 ^a,b^	7.9 ^a^	6.8 ^b^	7.1 ^b^	7.5 ^a^	7.2 ^b^	0.03

^1^ The most conservative standard error of the mean is presented. ^a^^–f^ Least squares mean with different superscript within variables among treatments across time, differ (*p* < 0.05; PROC GLM, PDIFF option). Note: Measurement nodes correspond to the experimental period as follows: Initial (Day 0), Middle (Day 30), and Final (Day 63).

**Table 5:** Least square means of the treatment × time interaction of reproductive and morphometric-testicular variables in Black Belly sheep (n = 12) supplemented with *Withania somnifera* (WITH; 400 mg kg^−1^ LW d^−1^), *Lepidium meyenii* (LEPI; 400 mg kg^−1^ LW d^−1^), or non-supplemented (CONT) at different stages of the experimental period (May–July, 64 d) in northern Mexico (25° N).

Variables ^1^	WITH		LEPI		CONT		SEM ^2^	*p*-Value
	Initial	Final	Initial	Final	Initial	Final		
ODOR (units)	0.0 ^c^	2.0 ^a^	0.0 ^c^	1.6 ^a^	0.0 ^c^	0.5 ^b^	0.1	0.01
SC (cm)	34.5 ^a^	34.3 ^a^	33.5 ^b^	34.0 ^b^	34.0 ^b^	33.5 ^b^	0.81	0.03
WIDTH-LFT (cm)	4.63 ^b^	5.52 ^a^	5.07 ^ab^	4.56 ^b^	5.63 ^a^	5.25 ^ab^	0.28	0.04
WIDTH-RIG (cm)	5.50 ^a^	4.92 ^a^	4.32 ^b^	4.22 ^b^	4.46 ^b^	4.24 ^b^	0.33	0.04
HEIGHT-LFT (cm)	8.73 ^a^	9.17 ^a^	9.28 ^a^	9.38 ^a^	9.06 ^a^	9.25 ^a^	0.65	0.89
HEIGHT-RIG (cm)	9.62 ^a^	8.88 ^a^	8.16 ^a^	8.93 ^a^	9.16 ^a^	8.76 ^a^	1.04	0.78
TRANS-LFT (cm)	4.26 ^a^	4.62 ^a^	4.23 ^a^	4.50 ^a^	4.71 ^a^	4.50 ^a^	0.24	0.76
TRANS-RIG (cm)	4.67 ^a^	5.19 ^a^	4.60 ^a^	3.99 ^a^	4.39 ^a^	3.70 ^a^	0.52	0.53
TVOL-LFT (cm^3^) ^3^	92.19 ^a^	122.3 ^a^	103.4 ^a^	100.4 ^a^	127.2 ^a^	108.1 ^a^	11.4	0.86
TVOL-RIG (cm^3^)	127.4 ^a^	116.4 ^a^	95.3 ^b^	79.6 ^b^	93.6 ^b^	71.4 ^b^	18.1	0.04
TTVOL (cm^3^)	219.6 ^a^	238.6 ^a^	198.7 ^b^	180.1 ^b^	220.8 ^a^	179.6 ^b^	19.8	0.04
EDSP (millions) ^4^	2716.2 ^a^	3277.4 ^a^	1927.3 ^b^	1678.3 ^b^	2720.5 ^b^	1856.4 ^b^	262.4	0.03

^1^ Sexual odor (ODOR, units); scrotal circumference (SC, cm), testicular volumetry (length (WIDTH, cm); height (HEIGHT, cm); depth (TRANS, cm) for left (LFT) and right (RIG) testicles, individual (i.e., TVOL cm^3^, left and right), and total testicular volumes (TTVOL cm^3^), as well as the estimated daily sperm production (EDSP, millions). ^2^ The most conservative standard error of the mean (SEM) is presented. ^3^ The calculation of the individual testicular volume (cm^3^) considered the formula 0.61 [width × height × trans] (i.e., TVOL; LFT and RIG). The sum of both generated the average total testicular volume (TTVOL); the epididymis was not included in the measurement. ^4^ The estimated daily sperm production (millions) considered the linear regression EDSP (M) = [(0.02226 × TTVOL) − 2.977] × 1000. ^a–c^ Least square means with different superscripts within variables among treatments differ (*p* < 0.05; PROC GLM, PDIFF option). Note: Measurement nodes correspond to the experimental period as follows: Initial (Day 0) and Final (Day 63).

## Data Availability

The original contributions presented in this study are included in the article. Further inquiries can be directed to the corresponding author.

## Abbreviations

The following abbreviations are used in this manuscript:

ANOVA	Analysis of variance
AOAC	Association of Official Analytical Chemists
BCS	Body condition score
BW	Body weight
CASA	Computer-assisted sperm analysis
CONT	Not supplemented
DM	Dry matter
DMI	Dry matter intake
DNA	Deoxyribonucleic acid
EDSP	Estimated daily sperm production
FAO	Food and Agriculture Organization of the United Nations
FSH	Follicle-stimulating hormone
GLU	Blood glucose
GnRH	Gonadotropin-releasing hormone
HEIGHT	Testicular height view
HEIGHT-LFT	Testicular height view, left
HEIGHT-RIG	Testicular height view, right
HO-1	Heme oxygenase-1
HPA	Hypothalamic–pituitary–adrenal axis
HPG	Hypothalamic–pituitary–gonadal axis
LEPI	*Lepidium meyenii*
LFT	Left
LH	Luteinizing hormone
LW	Live weight
MC	Milked cow
MUSD	Millions of United States dollars
NEL	Net Energy for Lactation
NF-κB	Nuclear factor kappa B
Nrf2	Nuclear factor erythroid 2-related factor 2
ODOR	Sexual odor
PRO	Serum protein
RIG	Right
SC	Scrotal circumference
SE	Standard error
T	Time
TMR	Total Mixed Ration
TRANS	Testicular transverse view
TRANS-LFT	Testicular transverse view, left
TRANS-RIG	Testicular transverse view, right
TRT	Treatment
TTVOL	Total testicular volume
TVOL	Testicular volume
TVOL-LFT	Testicular volume, left
TVOL-RIG	Testicular volume, right
WIDTH	Testicular length view
WIDTH-LFT	Testicular length view, left
WIDTH-RIG	Testicular length view, right
WITH	*Withania somnifera*
